# Anæsthetics and Accidents

**Published:** 1889-12

**Authors:** 


### Anæsthetics and Accidents.

A few weeks ago a well-known citizen of Philadelphia, in the prime of life, died suddenly after having been anæsthetized with nitrous oxide gas, in order to have a tooth extracted painlessly. He did not die while under the anæsthetic; but, soon after he recovered consciousness he had symptoms of apoplexy which increased until he died, after a lapse of a few hours. He was a man of large frame, of excellent general health and vigor, and of unexceptionable habits of life. The Coroner's investigation which followed did not include an autopsy, and so the immediate cause of death was not certainly known, but the diagnosis of apoplexy seems to have been justifiable.

This is an extraordinary case—and fortunately a rare one—of death following soon after anæthesia with nitrous oxide gas. No one, we believe, has asserted that in this case the anæsthetic directly caused death, and the general opinion seems to be that it was a mere coincidence. This opinion we can not agree with unconditionally; for we can not but believe the condition of nitrous oxide anæsthesia started the train of changes which culminated in apoplexy—if this was really the immediate cause of death.

Still, it may be said that accidents following anæsthesia with nitrous oxide gas are almost as rare as phœnixes, and scarcely to be named in contrast with its beneficent effects. The same may be said of ether and chloroform; although in their case danger and death are by no means uncommon.

Justice to the truth requires that all the circumstances attending a case of death, during or soon after anæsthesia, should be known and duly estimated before deciding in how much the result is fairly chargeable to the anæsthetic. In a case reported in the *British Medical Journal*, October 5, 1889, a woman in

Birmingham, twenty-five years old, of very nervous and excitable temperament, desired to have some teeth extracted, and insisted upon being anæsthatized. In the presence of her husband and the dentist, her medical attendant administered chloroform. The patient was seated in an easy chair, and, after inhaling a few breaths of chloroform, she slipped down in the chair, and her pulse and breathing stopped. Artificial respiration was at once resorted to, but without success. It seems perfectly clear that the patient was not anæsthetized when she died, as she had only just commenced to inhale the chloroform, and, of course, no attempt had been made to extract her teeth. The Coroner's jury returned a verdict that death was due to syncope, and that no blame whatever was attached to the administrator. In commenting on this case our British contemporary very truly says that it is well known that syncope may result from violent emotion, and especially from the effects of fear. It repeats the story of a case recorded in Germany a few years ago in which a female patient visited a dentist, and requested him to extract some carious teeth, demanding, at the same time, that she should be chloroformed. The dentist explained the risks of chloroform, and suggested nitrous-oxide gas; but his patient persisted, and he pretended to humor her Having, however, a wholesome dread of chloroform, he substituted Cologne water, and bade her inhale the supposed anæsthetic from a folded towel. After two or three inhalations she suddenly fell from the chair and died.

Such stories as this indicate that it would be but hasty and ill-considered judgment to look no further than to the anæsthetic to explain deaths which at first sight might seem chargeable to it. They indicate at the same time dangers connected with the administration of anæsthetics which are all the more serious because they are rarely encountered, and perhaps never anticipated. Due consideration of them, therefore, may serve a double purpose—in diminishing, as well as defining, the elements of real danger in anæsthesia.

A new soft alloy, which adheres so firmly to metallic, glass, and porcelain surfaces that it can be used as a solder, and which, in fact, is invaluable when the articles to be soldered are of such a nature that they can not bear a high degree of temperature, consists of finely pulverized copper dust, which is obtained, according to *Iron*, by shaking a solution of sulphate of copper with granulated zinc. The temperature of the solution rises considerably, and the metallic copper is precipitated in the form of a brownish powder; twenty, thirty, or thirty-six parts of this copper dust, according to the hardness desired, being placed in a cast-iron or porcelain-lined mortar and well mixed with some sulphuric acid, having a specific gravity of 1.85. To the paste thus formed are added seventy parts by weight of mercury, with constant stirring; and, when thus thoroughly mixed, the amalgam is well rinsed in warm water to remove the acid, and then set aside to cool. In ten or twelve hours it is hard enough to scratch tin. On being used, it is heated to a temperature of 375° C., and when kneaded in an iron mortar, becomes as soft as wax. In this ductile state it can be spread upon any surface, to which, as it cools and hardens, it adheres with great tenacity.

